# Numerical Investigation of Fatigue Behavior in Ti-6Al-4V Orthopedic Hip Implants Subjected to Different Environments

**DOI:** 10.3390/ma17153796

**Published:** 2024-08-01

**Authors:** Tamara Smoljanić, Ljubica Milović, Simon Sedmak, Aleksa Milovanović, Katarina Čolić, Zoran Radaković, Aleksandar Sedmak

**Affiliations:** 1 Innovation Centre of the Faculty of Mechanical Engineering, Kraljice Marije 16 Street, 11120 Belgrade, Serbia; tamaramijatovic1985@gmail.com (T.S.); amilovanovic@mas.bg.ac.rs (A.M.); kbojic@mas.bg.ac.rs (K.Č.); 2 Faculty of Technology and Metallurgy, University of Belgrade, Karnegijeva 4 Street, 11120 Belgrade, Serbia; acibulj@tmf.bg.ac.rs; 3 Faculty of Mechanical Engineering, University of Belgrade, Kraljice Marije 16 Street, 11120 Belgrade, Serbia; zradakovic@mas.bg.ac.rs (Z.R.); asedmak@mas.bg.ac.rs (A.S.)

**Keywords:** Ti-6Al-4V alloy, hip implants, fatigue crack growth, corrosion, Extended Finite Element Method

## Abstract

In this paper, hip implants made of Ti-6Al-4V titanium alloy are analyzed numerically using Extended Finite Element Method XFEM. The combined effect of corrosion and fatigue was considered here since this is a common cause of failure of hip implants. Experimental testing of Ti-6Al-4V alloy was performed to determine its mechanical properties under different working environments, including normal, salty, and humid conditions. The integrity and life of the hip implant were assessed using the Linear Elastic Fracture Mechanics (LEFM) approach. For this purpose, the conditional fracture toughness Kq using CT specimens from all three groups (normal, humid, salty conditions) were determined. This provided insight into how different aggressive environments affect the behavior of Ti-6Al-4V alloy; i.e., how much its resistance to crack growth would degrade depending on conditions corresponding to the real exploitation of hip implants. Next, analytical and XFEM analyses of fatigue behavior in terms of the number of cycles were performed for all three groups, and the obtained results showed good agreement, confirming the validity of the integrity assessment approach shown in this work, which also represented a novel approach since fatigue and corrosion effects were investigated simultaneously.

## 1. Introduction

Fatigue crack growth (FCG) is a major problem in the use of hip implants due to the high sensitivity of alloys used for its manufacturing, as shown in a couple of recent papers [1,2,3,4,5,6,7,8,9,10]. The three most commonly used materials in biomedical applications, 316L stainless steel, CoCrMo superalloy, and titanium alloy Ti-6Al-4V, are compared in terms of fatigue behavior in [11]. Out of these three, Ti-6Al-4V is considered here due to its exceptional corrosion resistance, mechanical properties, including elasticity modulus closest to the natural bone, and biocompatibility. Some aspects of the fatigue behavior of hip implants, made of Ti-6Al-4V or similar alloys, are presented in previous papers [1,2,3,4]. Design aspects of hip implants made of Ti-6Al-4V Extra Low Interstitials (ELI) alloy were considered in [1], with a focus on neck size, while its integrity assessment was presented in [2]. Experimental and numerical investigation of hip implant behavior under different exploitation conditions is presented in [2], proving the high capacity of Ti-6Al-4V alloy to withstand high loading. Numerical modeling of hip implants was also presented in [3], focused on static loading, as the basis of FCG analysis. A more focused numerical analysis of FCG of hip implants was presented in [4].

A detailed report on the fatigue behavior of Ti-6Al-4V can be found in the paper by Hosseini [5], along with explanations about why this alloy is considered one of the best materials for biomedical applications. In addition to good biocompatibility, it was determined that this alloy possesses fatigue strength similar to that of steel, which explains why it often replaces stainless steel as a material for implants. Geometry effects were also taken into account, by analyzing fatigue behavior around notches and other stress concentrators in implants. More details on geometry effects on fatigue resistance can be found in a paper by Hussenbocus et al. [6], which observes the neck–head connection in prosthetic hip implants along with the effects of cross-section reduction between the neck and stem on a more general level than in already mentioned research presented in [1,2]. Paper [6] showed also how fretting corrosion can occur in the neck–head interface, decreasing the fatigue life of hip implants. A similar analysis of hip implants with varying taper neck lengths under static loading conditions was presented in [7], based on the Finite Element Method (FEM) calculations.

An extensive review of wear and loading effects on materials for hip prostheses is given in [8]. Experimental and numerical investigation of Ti-6Al-4V alloy behavior under different exploitation conditions was presented in [9]. An excellent review of the design evolution of hip implants is given in [10]. Although used for different purposes (orthopedic plates), fatigue life assessment of Ti-6Al-4V, based on experimental and numerical analysis, represents another useful source of information [12,13,14,15]. The fatigue life was calculated by using the extended FEM (XFEM) in combination with experimental results. The referenced work did not take corrosion into account, but it included different versions of geometry, illustrating its considerable effects on the performance of the plates.

As for the research performed before the one presented here, numerical simulation via the finite element method of Ti-6Al-4V can be found in [16,17,18,19]. These papers were mainly focused on the numerical analysis of fatigue effects but did not involve corrosion and only a few included experimental analyses. Corda et al. [20] presented fatigue life evaluation of different hip implant designs using finite element analysis, while Zameer et al. [16] provided fatigue life estimation of artificial hip joints using FEM. Experimental work related to fracture mechanics and Ti-6Al-4V alloy was presented in [17,18]. Tsay et al. [17] investigated the effects of welding defects on the integrity of structures made of this alloy. Nader et al. [18], on the other hand, used a probabilistic approach to determine the fatigue resistance of various metallic materials, including Ti-6Al-4V. These rare examples of work involving fracture mechanics of structures made of Ti-6Al-4V alloy offer some very interesting results but do not include the effects of corrosion.

One of the earliest examples of investigating a combination of fatigue and corrosion in Ti-6Al-4V implants was given in [19]. This paper deals with titanium specimens being subjected to rotation cyclic loads, while also observing the changes in corrosion potential. While this research considers fatigue in the form of S-N curves, it still confirms the excellent fatigue behavior of the alloy in question—a very high number of cycles was achieved for loads whose magnitudes by far exceed the ones that occur in human bodies, even during more extreme load cases, such as running. Failure analysis of three uncemented titanium-alloy modular total hip implants is presented in [21], indicating the occurrence of a fatigue process accompanied by corrosion. It was shown that the presence of numerous latent microcracks demonstrated additional embrittlement of implant structure assisted by the hydrogen environment. On the other hand, one of the most recent research projects on the effects of corrosion on FCG of Ti-6Al-4V ELI titanium alloy is presented in [22], although it is intended for different applications.

Having in mind the importance of corrosion, research in this paper is based on experimental, analytical, and numerical analysis to determine the effects of a human body on the fatigue behavior of the Ti-6Al-4V alloy hip implant. This topic is of particular interest since it was not addressed previously in such a systematic way. Based on the above information about previous work on this topic, it can be concluded that the approach adopted for this analysis provides original results including the experimental determining of equivalent stress intensity factor (hereinafter denoted as K_q_) of titanium alloy subjected to different environments.

## 2. Experimental Determination of Ti-6Al-4V Mechanical Properties

To obtain the necessary data for the numerical simulations, several experiments were performed, some of which are described in more detail in [2]. Since the goal here was to determine how different environments affect the mechanical properties, tensile test specimens made of Ti-6Al-4V were made for three different testing conditions:

- The first group included specimens that were not subjected to any aggressive environment, i.e., were kept in common air (code name-ZA-1, ZA-2, and ZA-3).
- Second group of specimens, which were kept in a salty environment (code name-ZS-1, ZS-2, and ZS-3).
- Third group of specimens, which were kept in a humid environment (code name-ZV-1, ZV-2, ZV-3).

**Figure 1:**
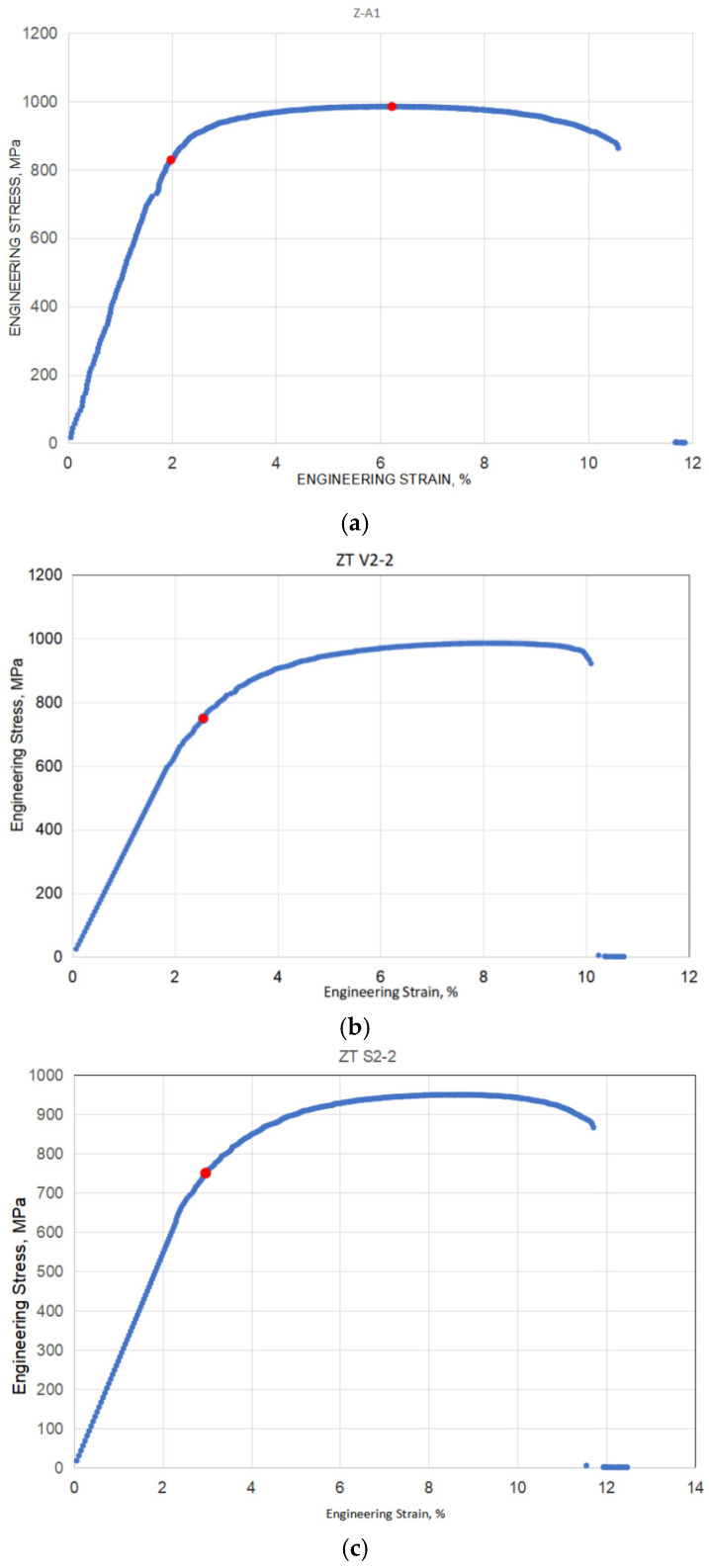
Stress–strain diagrams from the tensile test for all three groups: (**a**) dry; (**b**) humid, (**c**) salty.

Specimens from the humid and salty conditions groups were kept in Weiss 206553/8/0001/S 100 SCC chambers for a period of 30 days, to simulate the corrosive environments that are typically encountered in the human body. Salty conditions were simulated by keeping the specimens in this chamber for 2 h, followed by 22 h in a salty mist atmosphere, and for the other specimens, humid atmosphere was used (with the same chamber as previously mentioned). This cycle was repeated for a total of 30 days. All specimens were photographed and degreased using alcohol before being placed in the chamber. Visual inspection was performed after each test. All tensile tests were performed using an INSTRON test machine with a load capacity of 25 tonnes. Results obtained by tensile tests are shown in Table 1. More details about this part of the experimental research can be found in [9]. Examples of the stress–strain diagrams that were obtained during the experiment can be seen in Figure 1 (one diagram for each group).

From a statistical point of view, one should notice average values and deviations for tensile properties, as given in Table 1. In the case of Yield stress, R_p0.2_, average values are 825 (+31/−33) MPa for normal conditions, 755.7 (+7.3/−5.7) MPa for salty conditions, and 769.7 (+30.3/−20.7) MPa for humid conditions, indicating deviations less than 4% (in normal and humid conditions), and less than 1% (in salty conditions). In the case of Tensile strength, Rm, average values are 984.7 (+1.3/−1.7) MPa for normal conditions, 940 (+10/−8) MPa for salty conditions, and 971.7 (+13.3/−6.7) MPa for humid conditions, indicating deviations less than 0.15% (in normal), 1.1% (in salty conditions), and less than 1.5% (in humid conditions). In the case of Elongation, A, average values are 12.9 (+3.3/−2.3) % for normal conditions, 12.2 (+0.6/−0.5) % for salty conditions, and 11.6 (+1.2/−1.5) % for humid conditions, indicating deviations of 25% (in normal), 5% (in salty conditions), and 10% (in humid conditions). One can see that deviations are the highest in the case of Elongation, and lowest in the case of Tensile strength.

**Figure 2:**
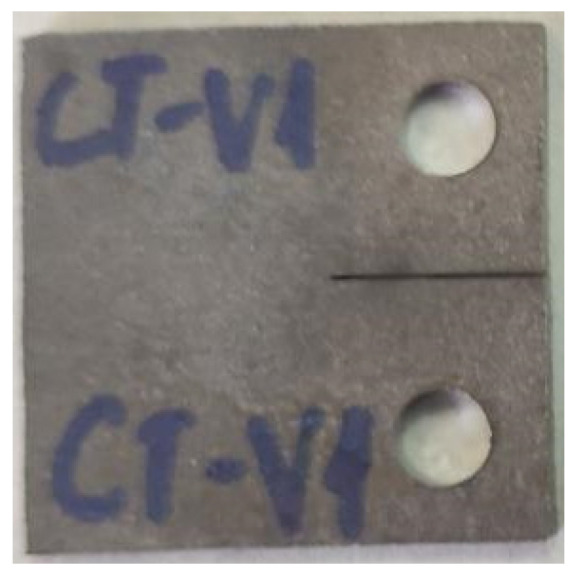
An example of a CT specimen (code name, CT-VT1).

For the fracture toughness measurement, Compact Tension (CT) specimens were used, taken from the available material thickness, 2.2 mm, whereas the width was 33.6 mm and the initial crack length was 17.3 mm. An example of CT specimens is shown in Figure 2, marked as CT-V1 (humid condition, specimen No. 1). Other CT specimens were marked in the same way as tensile specimens, i.e., CT-A1/2/3 for normal, CT-S1/2/3 for salty, and CT-V1/2/3 for humid condition.

**Figure 3:**
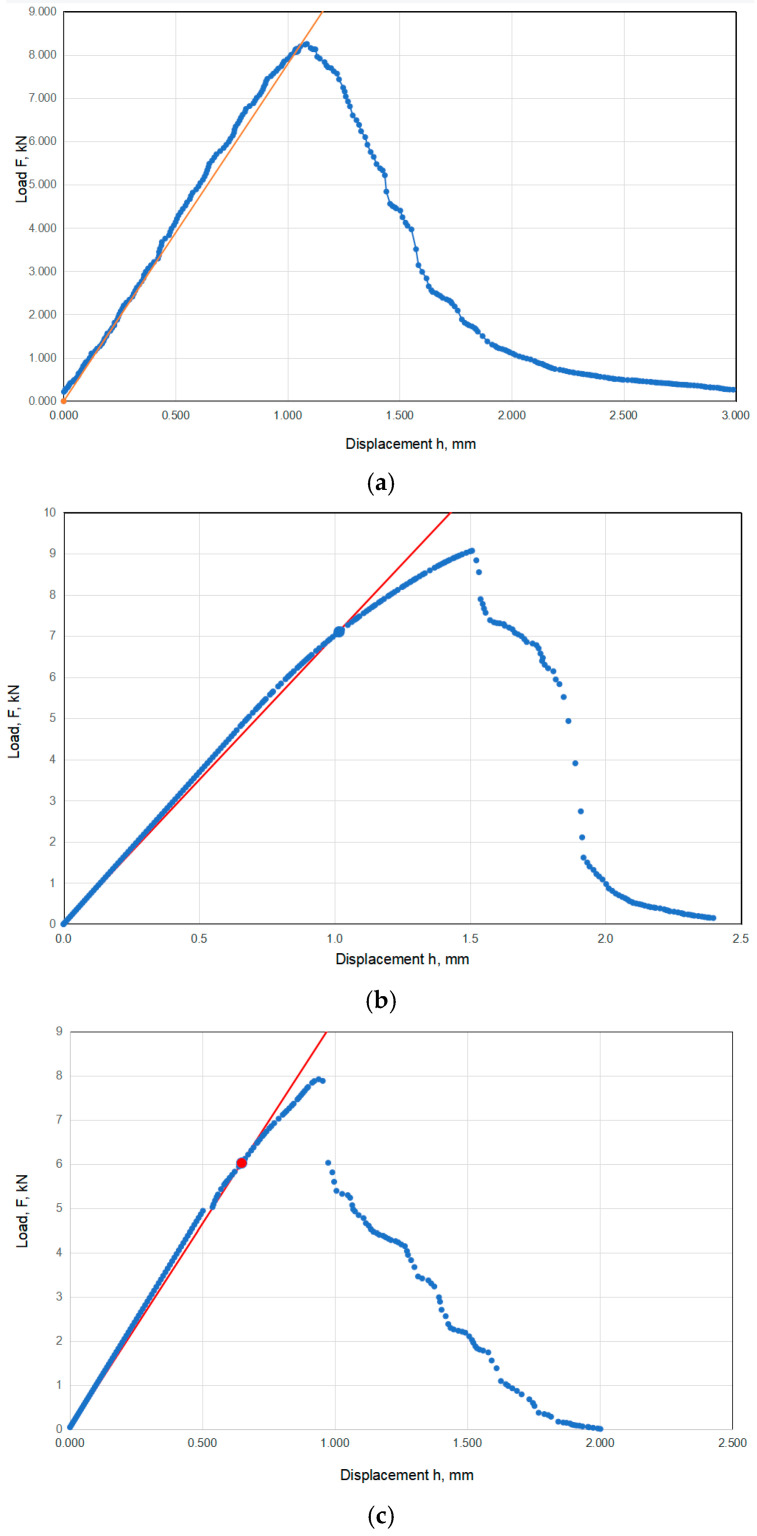
Force–displacement diagrams for specimens: (**a**) CT-A1, (**b**) CT-S2, (**c**) CT-V1.

Representative force–displacement diagrams are shown in Figure 3, one for each group (normal, humid, and salty conditions). Conditional fracture toughness *K_q_* is calculated according to the following formulas [23,24,25]:

(1) $$K_{q}=\frac{P}{BW^{1/2}}f\left(\frac{a}{W}\right)$$

where *P* is the force at yield stress, *B* is the thickness of the specimen, *W* is the width (33.6 mm), *a* is the crack length, and *f* is the geometry factor, depending on the *a*/*W* ratio.

The standard procedure also requires to check the plane strain condition, i.e., the minimum specimen thickness *B*_min_:

(2) $$B_{\min}=2.5\cdot {\left(\frac{K_{q}}{\sigma_{YS}}\right)}^{2}$$

Using Equations (1) and (2), *K_q_* and *B*_min_ values are calculated and shown in Table 2. As expected, the thickness of the specimens (2.2 mm) was far beyond the minimum standard requirement. However, since the purpose of this experiment was to compare the fracture behavior of Ti-6Al-4V alloy in different environmental conditions, this was not relevant to our analysis.

From the above table, the average values for normal, salty, and humid specimens were determined for the conditional fracture toughness, *K_q_*: 195.5 (+32.8/−30.6) MPa·m^1/2^ for normal conditions, 148.1 (+30.1/−20.9) MPa·m^1/2^ for salty conditions, and 166.4 (+11.4/−15.4) MPa·m^1/2^. for humid conditions, indicating deviations of 17% (in normal conditions), and less than 22% (in humid conditions) and 7% (in salty conditions).

### Fatigue—Paris Law

**Figure 4:**
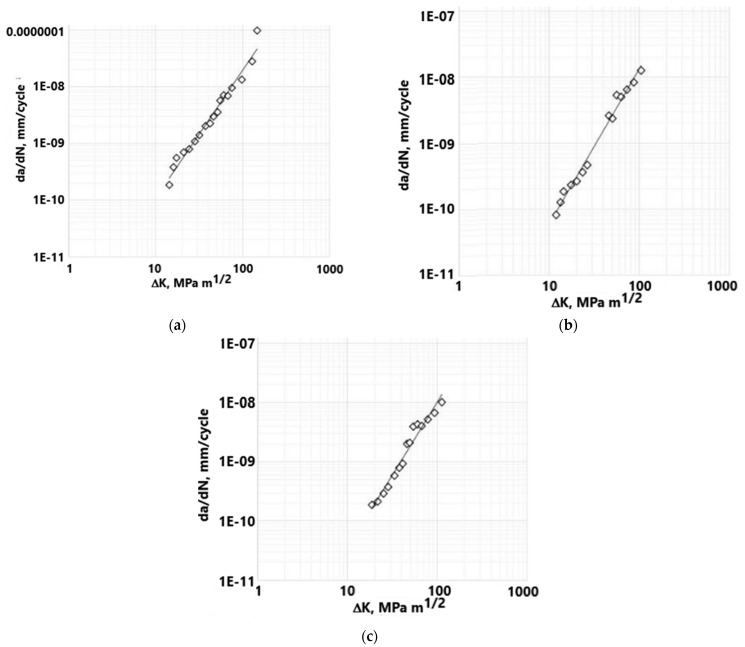
Diagrams da/dN-ΔK for (**a**) normal, (**b**) humid, (**c**) salty conditions.

In this case, Paris law coefficients C and m were experimentally determined, according to the Standard Test Method for the Measurement of Fatigue, ASTM E647 [26]. A RUMUL Fractomat (Russenberger prufsmachinen, Neuhausen, Switzerland) device was used for this experiment, with standard Charpy specimens (10 × 10 × 55 mm in bulk). The testing frequency was around 100 Hz, and the initial stress intensity threshold was around 10 MPa·m^1/2^. The load ratio was defined as R = 0, corresponding to a typical load/unload cycle during walking and other physical activities to which hip implants are subjected in everyday use. The following values were obtained: C = 6.72 × 10^−13^ and m = 2.26 for the normal conditions, C = 5.3 × 10^−13^ and m = 2.75 for the humid conditions, and C = 1 × 10^−12^ and m = 2.47 for salty conditions. All of the previously mentioned values are expressed in mm/cycle. These values were obtained experimentally, based on the da/dN vs. ΔK diagrams for all three cases, see Figure 4. The figure below shows the representative diagrams since a total of three specimens were tested for each group. The Paris coefficient combinations that were adopted are the least favorable ones from each group, to ensure a conservative approach.

## 3. Analytical Calculation

Analytical calculation of the number of cycles needed for a crack to grow from the initial to the final value is performed according to the integral form of the Paris law:

(3) $$N=\frac{1}{\left(\frac{m-2}{2}Cf^{m}\pi^{\frac{m}{2}}\cdot \Delta \sigma^{m}\right)}\cdot \left[\frac{1}{a_{0}^{\frac{m-2}{2}}}-\frac{1}{a_{c}^{\frac{m-2}{2}}}\right]$$

where *N* is the number of cycles, *C* and m are Paris law coefficients, and *f* is the geometry factor, depending on *a*/*W*. In the case analyzed here, *a*_0_ = 1 mm, taken as the depth of the circumferential surface crack. Therefore, the analytical calculation was conservative since the crack is treated as an edge crack growing into structural depth.

The next step involved the calculation of critical crack length *a_c_*, based on a well-known relation [25]:

(4) $$a_{c}=\frac{1}{\pi }{\left(\frac{Kq}{\sigma_{\max}\cdot f}\right)}^{2}$$

where *f* once again represents the geometry factor, which is 1.12 for the short cracks (*a*/*W* less than 0.1) and increases with increasing *a*/*W* value. This means that one does not know its value since *a_c_* is not known, requiring an iterative procedure to estimate *a_c_*. Taking into account average values of *K_q_* for different conditions (normal *K_q_* = 195.5 MPa·m^1/2^, salty 148.1 MPa·m^1/2^, and humid *K_q_* = 166.4 MPa·m^1/2^) and unique values for *f* (i.e., 1.12) and σmax (175.5 MPa, as shown in [1]), one obtains values for a_c_ that are significantly larger the neck thickness (W = 14.6 mm). Therefore, *a_c_* was determined by iterative procedure with increasing *f* values, and the results are shown in Table 3.

Taking into account values for *a_c_*, as well as C and m, the total number of cycles for different conditions was calculated by a simple incremental procedure with crack size increments of 1 mm and values *f* taken at the mid-increment crack sizes (1.5, 2.5, 3.5 mm, etc.). Results for a cycle number are also shown in Table 3, for all three conditions.

## 4. Numerical Simulations of Fatigue Crack Growth in Ti-6Al-4V Hip Implants

**Figure 5:**
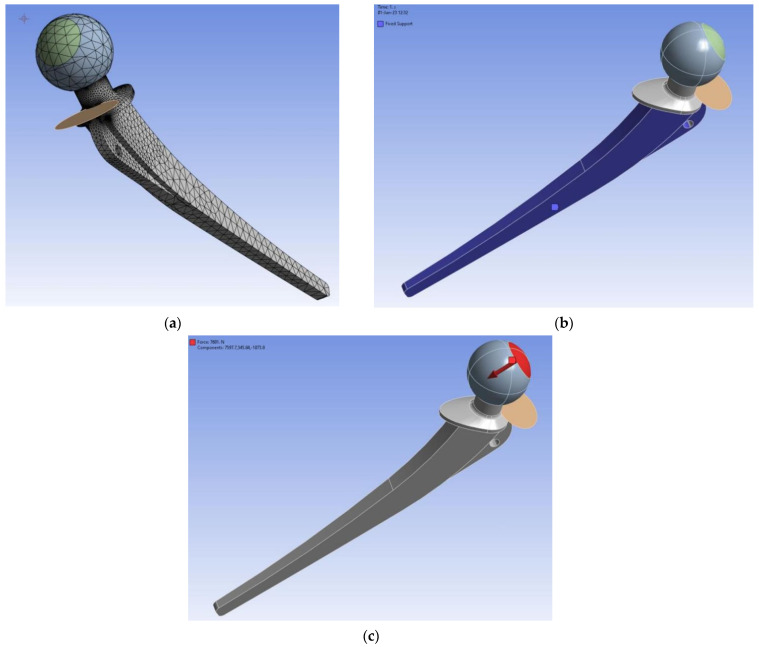
(**a**) Finite element mesh, (**b**) boundary conditions—blue, (**c**) applied load—red.

Numerical simulation of fatigue crack growth is based here on the Extended Finite Element Method (XFEM), a widely used tool in numerous scientific and industrial fields, as shown in a couple of examples [27,28,29,30]. For this research, ANSYS 2022R2 software was used in order to simulate fatigue crack growth. The geometry of the numerical models, along with the appropriate load due to normal walking, boundary conditions (fixed along the stem), and the finite element mesh are shown in Figure 5. As can be seen in Figure 5, the fixed boundary condition, which prevents displacement and rotations along all three axes, is denoted by the blue color. This boundary condition corresponds to a real hip implant being positioned inside the bone. The load was determined to be 7681 N (see Figure 5c), based on the literature and previous experience [1,2,3,4], corresponding to a maximum stress of 175.5 MPa. An initial, penny-shaped crack was located in the hip implant’s neck area, due to the highest stress concentration there, as shown in [1,2,3,4]. The neck width was 14.6 mm, which ensures longer structural life, but on the other hand, decreases the patient’s mobility. Other options for neck width were analyzed in [2].

An overview of the most important parameters for fatigue crack growth simulations in ANSYS is given in Table 4. Yield stress and tensile strength values were determined as averages for three specimens from each group.

**Figure 6:**
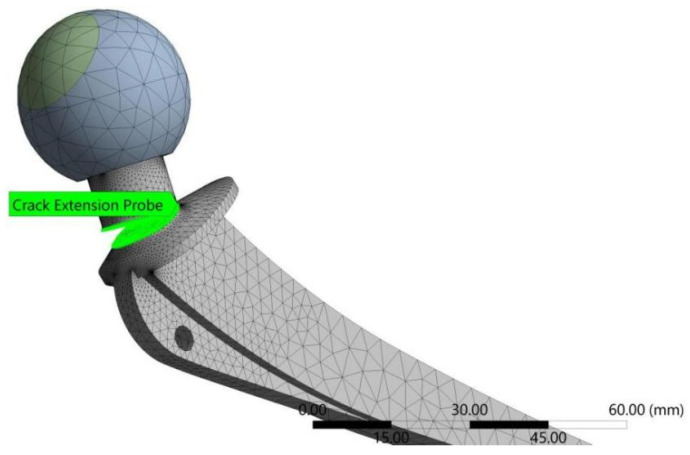
Hip implant model (normal conditions) after the critical crack length of 9.9 mm was reached.

**Figure 7:**
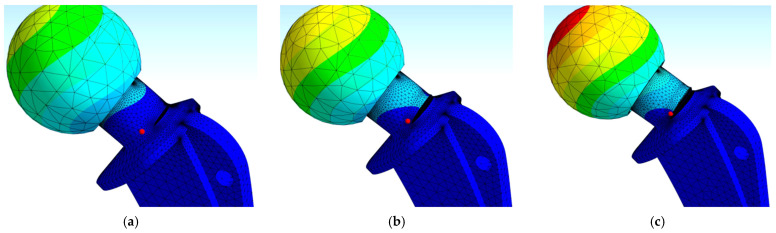
Fatigue crack growth: (**a**) initiation, (**b**) stable growth, (**c**) unstable growth.

Simulations involved three different sets of input data for three environmental conditions. The goal of these simulations was to observe the total number of cycles for all three conditions and to compare them to the analytically obtained ones. For this purpose, each model had a specific number of substeps defined to ensure that the proper crack lengths, taken as the dimension along the depth, were achieved as the final result. Figure 6 shows the deformed hip implant model (normal conditions) after the required crack length was achieved, indicating the exceptional resistance of a Ti-6Al-4V material to FCG. Figure 7a–c show a more detailed view of FCG during various stages of loading. The FCG was very similar in all three cases, in terms of the plane of propagation, with the main difference being in the final crack length. In all three cases, the crack propagated through the plane adjacent to the sudden change in geometry, i.e., the location of the highest stress concentration. Initial crack length was selected as 1 mm, since this is the standard minimum crack length that is considered detectable by surface non-destructive test methods.

Finite element mesh size was iteratively determined through a number of attempts, until a sufficiently convergent model was obtained. When generating a mesh in ANSYS, the software can automatically refine it in the area around the crack, and this option was used in order to obtain a very fine mesh around the critical area of the model. As for the fracture plane, it is also automatically determined during the simulation, and there was no need to manually set it.

**Figure 8:**
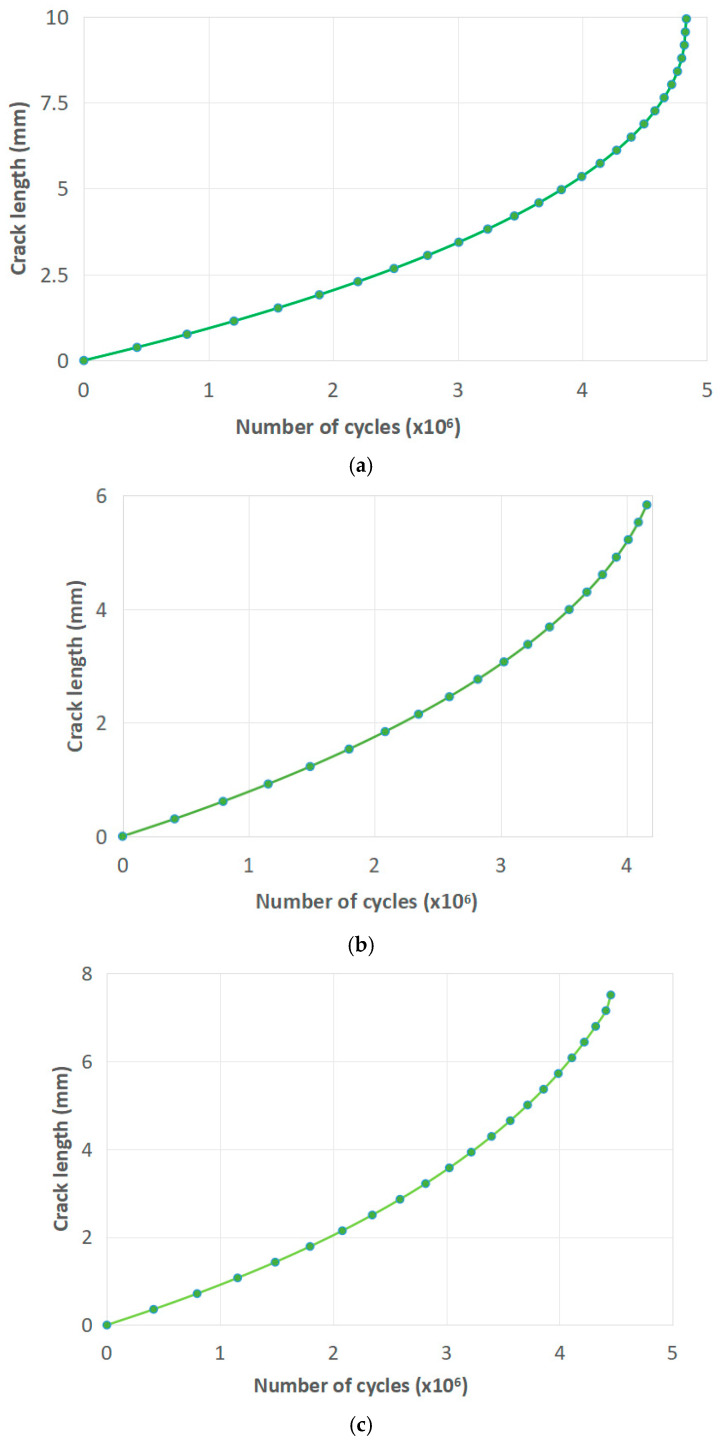
The a-N diagrams for (**a**) normal, (**b**) salty, (**c**) humid conditions.

Another factor to consider was if these numbers of cycles/crack lengths would lead to the failure of the hip implant, i.e., if the crack length vs. number of cycles diagrams (a-N) would reach the unstable FCG region of the Paris curve. Results of all three simulations for final crack lengths of 5.8, 7.3, and 9.9 mm are shown in Figure 7a–c. As one can see from Figure 8, the number of cycles is 4,869,300, 4,450,800, and 4,135,900 for normal, humid, and salty conditions, respectively.

## 5. Discussion

As expected, salty and humid conditions reduced tensile properties (salty YS 8.4%, TS 4.6%, Elong. 5.5%, humid YS 6.7%, TS 1.3%, Elong. 10%), although not significantly. The small degree of differences can be easily explained by the exceptional resistance of Ti-6Al-4V to corrosion. One should also notice that deviations from the average values of tensile properties are the same or smaller in salty and humid conditions than in normal conditions, leading to the same conclusion.

Results obtained for relevant fracture mechanics parameters (*K_q_* in this case, as already explained) indicate similar behavior, with more pronounced differences: humid conditions had *K_q_* values around 15% lower than the first (normal) group, salty conditions specimens showed the lowest values of *K_q_*, being around 11% lower than the humid group and 25% lower compared to the normal specimens. Out of all three groups, salty specimens showed the most consistent results, without extremely high or low values, like CT-A3 and CT-S2. One can say that the results for the relevant fracture mechanics parameters also prove the high corrosion resistance of Ti-6Al-4V alloy but with more expressed sensitivity to cracks than to tensile properties.

FCG was also affected more by salty, than by humid, conditions, presumably not as much as fracture toughness. The effect of humid and salty conditions was more pronounced on critical crack lengths, with normal conditions specimens reaching lengths almost two times greater than that of the salty specimens (9.98 vs. 5.8 mm) and humid specimens being between these two values (7.25 mm). The number of cycles to reach the critical crack length from the initial 1 mm length was not that much different, ranging from 3,789,105 (in salty) and 4,134,232 (in humid) to 4,447,126 (in normal conditions) in analytical calculation, with similar, but ca. 10% higher values in numerical simulation (4,135,900 for salty, 4,450,800 for humid, and 4,869,300 for normal conditions). Numerical simulations have shown similar behavior in terms of differences between the three groups, which was expected since the input material parameters were the same (C and m). A somewhat larger number of cycles was also expected, since the analytical calculation was more conservative due to the representation of the crack, which was a 2D edge, compared to a 3D surface crack in XFEM.

Although the final comparisons have shown very good agreement, there are still some questions to be addressed. The numerical analyses shown here only involved one load case (regular walking); hence, more results could be obtained for other, more extreme types of loads. Research can be further improved by performing detailed fatigue testing via experiments, which were not performed here due to time and resource constraints. This would provide a more detailed set of input data, namely, the Paris law coefficients, which could help gain better insight into the behavior of titanium alloy hip implants subjected to corrosion. Finally, once this methodology is fully developed, verified, and improved, it could also be applied to a wide variety of other materials, as well as to applications other than biomedicine.

This research also involved the combining of two different fracture mechanics principles, due to limitations related to available experimental tests—CT specimens had dimensions that caused a plane stress state in them during the tests, wherein numerical models included the actual geometry of a hip implant, with a much thicker neck as a critical location for fatigue crack initiation—i.e., numerical models had a plane strain state. As a consequence, values of fracture toughness for the experimental specimens had to be evaluated as *K_q_* and were significantly higher than what would have been obtained in the case where it was possible to use K_Ic_. However, keeping in mind that these results were used only for comparison, they were relevant for this research.

## 6. Conclusions

The research presented in this paper involved several different approaches to determining the behavior and fatigue life of Ti-6Al-4V hip implants subjected to different types of corrosion. These approaches included experimental determination of mechanical properties, including the conditional fracture toughness *K_q_*, analytical calculation of critical crack lengths and total numbers of cycles, and numerical simulations of fatigue crack growth. Based on the presented research, the following conclusions were drawn:

- Experimental, analytical, and numerical methods used for obtaining the results for fatigue crack growth showed good agreement with each other.
- The investigated titanium alloy, Ti-6Al-4V, has exceptional resistance to aggressive environments, typically encountered inside a human body, since even in the worst case (salty environment), the fatigue life decreased by less than 20%, and similar reduction for the fracture toughness can be estimated, based on *K_q_* evaluation.
- Proposed methodologies can be used effectively regardless of material, and can be further improved by using *K_Ic_* instead of *K_q_* (as the relevant fracture mechanics parameter).

Future research on this topic can include other failure mechanisms and their combined effects on fatigue crack growth, as well as different hip implant geometries and materials. In this way, the integrity of hip implants could be improved, resulting in a longer work life. An additional subject that could be considered includes the nucleation and initiation of fatigue cracks, which would provide a more detailed insight into the fatigue behavior of titanium alloy hip implants.

## Figures and Tables

**Table 1:** Tensile properties of Ti-6Al-4V for normal, salty, and humid conditions [9].

Specimen Condition	Mark	Yield Stress, R_p0.2_ (MPa)	Tensile Strength, Rm (MPa)	Elongation, A (%)
Normal	Z-A1	829	985	10.6
	Z-A2	792	983	16.2
	Z-A3	854	986	11.8
Salty	Z-S1	763	938	12.8
	Z-S2	750	950	11.7
	Z-S3	754	932	12.0
Humid	Z-V1	760	965	12.0
	Z-V2	749	985	10.1
	Z-V3	800	965	12.8

**Table 2:** Results for the conditional fracture toughness *K_q_* and minimum necessary thickness B.

Specimen	*K_q_* (MPa·m^1/2^)	*B*_min_ (mm)	Specimen Group
CT-A1	203.2	151.6	Normal
CT-A2	218.3	191.3	Normal
CT-A3	164.9	94.4	Normal
CT-S1	139.0	84.6	Salty
CT-S2	178.2	141.0	Salty
CT-S3	127.2	72.5	Salty
CT-V1	151.0	100.1	Humid
CT-V2	170.3	130.7	Humid
CT-V3	177.8	124.8	Humid

**Table 3:** Critical crack lengths and cycle number.

Environmental Conditions	*a_c_* (mm)	*N* (-)
Normal	9.98	4,477,126
Humid	7.25	4,134,232
Salty	5.8	3,789,105

**Table 4:** Input parameters for all three models.

Model	Yield Stress, R_p0.2_ (MPa)	Tensile Strength, Rm (MPa)	Paris Coefficients	
			C	m
Normal	825	985	6.72 × 10^−13^	2.26
Salty	756	940	5.3 × 10^−13^	2.75
Humid	770	972	1 × 10^−12^	2.47

## Data Availability

The original contributions presented in the study are included in the article, further inquiries can be directed to the corresponding author.
